# Needs and perceptions regarding healthy eating among people at risk of food insecurity: a qualitative analysis

**DOI:** 10.1186/s12939-019-1077-0

**Published:** 2019-11-27

**Authors:** Laura A. van der Velde, Linde A. Schuilenburg, Jyothi K. Thrivikraman, Mattijs E. Numans, Jessica C. Kiefte-de Jong

**Affiliations:** 1 Department of Public Health and Primary Care/ LUMC-Campus The Hague, Leiden University Medical Centre, The Hague, The Netherlands; 2 Faculty of Governance and Global Affairs, Leiden University College, The Hague, The Netherlands; 3 Department of Epidemiology, Erasmus University Medical Center, Rotterdam, The Netherlands

**Keywords:** Healthy eating, Eating behaviour, Food insecurity, Barriers, Food environment, Financial stress, Health, Mental health, Children

## Abstract

**Background:**

Healthy eating behaviour is an essential determinant of overall health. This behaviour is generally poor among people at risk of experiencing food insecurity, which may be caused by many factors including perceived higher costs of healthy foods, financial stress, inadequate nutritional knowledge, and inadequate skills required for healthy food preparation. Few studies have examined how these factors influence eating behaviour among people at risk of experiencing food insecurity. We therefore aimed to gain a better understanding of the needs and perceptions regarding healthy eating in this target group.

**Methods:**

We conducted a qualitative exploration grounded in data using inductive analyses with 10 participants at risk of experiencing food insecurity. The analysis using an inductive approach identified four core factors influencing eating behaviour: Health related topics; Social and cultural influences; Influences by the physical environment; and Financial influences.

**Results:**

Overall, participants showed adequate nutrition knowledge. However, eating behaviour was strongly influenced by both social factors (e.g. child food preferences and cultural food habits), and physical environmental factors (e.g. temptations in the local food environment). Perceived barriers for healthy eating behaviour included poor mental health, financial stress, and high food prices. Participants had a generally conscious attitude towards their financial situation, reflected in their strategies to cope with a limited budget. Food insecurity was mostly mentioned in reference to the past or to others and not to participants’ own current experiences. Participants were familiar with several existing resources to reduce food-related financial strain (e.g. debt assistance) and generally had a positive attitude towards these resources. An exception was the Food Bank, of which the food parcel content was not well appreciated. Proposed interventions to reduce food-related financial strain included distributing free meals, facilitating social contacts, increasing healthy food supply in the neighbourhood, and lowering prices of healthy foods.

**Conclusion:**

The insights from this study increase understanding of factors influencing eating behaviour of people at risk of food insecurity. Therefore, this study could inform future development of potential interventions aiming at helping people at risk of experiencing food insecurity to improve healthy eating, thereby decreasing the risk of diet-related diseases.

## Background

Healthy eating behaviour is an essential determinant of overall health. Previous literature extensively shows that people with lower socioeconomic status (SES) generally exhibit less healthy eating behaviours [1] and have increased risk of obesity and related illnesses [2, 3]. The same holds for people experiencing food insecurity [4–6], which is an inadequate physical and economic access to adequate food that meets dietary needs and food preferences [7]. The concept of food insecurity is closely related to lower SES, although this is a complex relationship and people with lower SES do not always experience food insecurity and vice versa [8]. However, it is evident that food insecurity is more common among people with lower SES and therefore people with lower SES or living in disadvantaged neighbourhoods have an increased risk of experiencing food insecurity [9].

Thus far, knowledge on food insecurity in Europe is limited [10]. A previous study among Dutch Food Bank recipients found a food insecurity prevalence of almost 73% [11]. Our recent study has shown that approximately one quarter of families living in disadvantaged neighbourhoods in The Netherlands experienced food insecurity (van der Velde LA, Nyns CJ, Engel MD, Neter JE, van der Meer IM, Numans ME, et al: Exploring food insecurity and obesity in Dutch families: a crosssectional mediation analysis, unpublished). Results of this study further showed that general health, diet quality, and weight were suboptimal, especially among food insecure participants. A possible intervention for reducing food insecurity is the Food Bank, but despite the high prevalence of food insecurity it was hardly used (van der Velde LA, Nyns CJ, Engel MD, Neter JE, van der Meer IM, Numans ME, et al: Exploring food insecurity and obesity in Dutch families: a crosssectional mediation analysis, unpublished). The Dutch Food Bank is a non-governmental organization that distributes donated food to offer temporal food aid to people in need [12]. This is done through providing food parcels, meant to supplement the usual diet, to eligible persons. Eligibility is based on household size-adjusted monthly disposable income. The food parcel content largely depends on donated foods and therefore varies per time and location of Food Bank. Recent research indicated that the parcel content was generally not in line with nutritional guidelines, which may contribute to suboptimal dietary intake among people eligible for Food Bank use [13].

Various factors may contribute to the generally suboptimal eating behaviour among people at risk of experiencing food insecurity, including stress [14–16], inadequate knowledge and skills regarding healthy eating and food preparation [17], and higher costs of healthy foods [18]. These higher costs might be an even more prominent issue than previously, since the Dutch Government recently increased taxes of all basic necessities such as foods (including foods that are considered healthy like fruit and vegetables) from 6 to 9% [19]. This price increase may lead to less healthy eating behaviour, as previous research shows that pricing affects food choices [20, 21].

Much uncertainty still exists about contributing factors to suboptimal eating behaviour among people at risk of experiencing food insecurity. Improving insight is essential for developing targeted interventions to support this population, focused on improving healthy eating behaviour and thereby decreasing diet-related disease risk. Therefore, we aimed to gain a better understanding of the needs and perceptions regarding healthy eating behaviour of people at risk of experiencing food insecurity living in disadvantaged neighbourhoods in the Netherlands.

## Methods

### Rationale and study sample

Participants were selected from a sample of 242 participants included in a cross-sectional study on food insecurity in disadvantaged neighbourhoods in The Hague, The Netherlands (van der Velde LA, Nyns CJ, Engel MD, Neter JE, van der Meer IM, Numans ME, et al: Exploring food insecurity and obesity in Dutch families: a crosssectional mediation analysis, unpublished). These neighbourhoods were selected based on predefined criteria used by the Dutch Government to identify disadvantaged neighbourhoods in the Netherlands [22]. Participants lived in or near the preselected disadvantaged neighbourhoods and had at least one child below the age of 18 years living at home. A detailed description of the methods and results of this study are described elsewhere (van der Velde LA, Nyns CJ, Engel MD, Neter JE, van der Meer IM, Numans ME, et al: Exploring food insecurity and obesity in Dutch families: a crosssectional mediation analysis, unpublished). Participants who provided valid contact information were invited to take part in an interview. None of the participants that agreed to participate dropped out of the study. Reasons for refusing to participate included being too busy, thinking an interview of approximately 60 min was too long, and being or going on holiday. A convenience sample, taking into account the diversity of the study sample, of a total of 10 participants (either fathers or mothers, one parent per household) were interviewed. After those 10 interviews, thematic saturation was reached. Interviews were conducted between April and July 2018. Sociodemographic characteristics, food insecurity status and diet quality scores of the participants were previously assessed (van der Velde LA, Nyns CJ, Engel MD, Neter JE, van der Meer IM, Numans ME, et al: Exploring food insecurity and obesity in Dutch families: a crosssectional mediation analysis, unpublished). Food insecurity status was assessed using the 18-item United States Department of Agriculture (USDA) Household Food Security Survey Module. Affirmative responses to the questions (described in Additional file 1: Table S1) were summed and resulted in a continuum of food insecurity status ranging from 0 to 18, categorized as ‘food secure’ (0–2 affirmative responses), and ‘food insecure’ (≥3 affirmative responses), according to the USDA standards [23, 24]. Dietary intake was assessed using the Dutch Healthy Diet Food Frequency Questionnaire (DHD-FFQ) [25]. Based on this dietary intake data we constructed a food group-based 6-component diet quality score (Additional file 2: Table S2). Each component score reflected the adherence to the dietary guidelines of the concerning food group. Component scores were summed to obtain the total diet quality score (range 0–60), with higher scores indicating a better diet quality. Written informed consent was obtained from all participants. Participants received a financial compensation of 10 euros for their effort and any travel expenses were refunded. The study was approved by the Medical Ethics Committee of Leiden University Medical Centre (P17.164).

### Study design

Face-to-face open interviews were conducted, guided by a topic list (Additional file 3: Table S3). The topic list was created at the start of the study based on issues raised in the previous study (van der Velde LA, Nyns CJ, Engel MD, Neter JE, van der Meer IM, Numans ME, et al: Exploring food insecurity and obesity in Dutch families: a crosssectional mediation analysis, unpublished) and consisted of topics to discuss and open ended example questions for each topic to guide the interviewer. These topics and example questions were discussed within the research team. The interviews started with general questions concerning participants’ background, family, and living conditions to make the participant feel at ease, followed by questions focusing on perceptions regarding healthy eating, including knowledge; skills; external, social, and cultural influences; health; finances; stress; environmental factors; opinions about eating on a low budget; existing resources; and Food Bank use. Interviewees were also free to introduce other topics that were of interest to them. The topic list was merely used as guidance during the interviews and was re-evaluated after each interview and if appropriate adjusted or complemented with new topics that emerged during the interview. During the interviews, two members of the study team were present; one of them conducted the interview and the other observed. All interviews were audio-recorded with participants’ permission using a digital voice recorder and transcribed verbatim. Participants were interviewed at a time and place that was most convenient to them. Interviews were held for 22 to 76 min with an average interview time of 47 min.

### Analysis

We used a general inductive approach to analyse the data [26]. Segments of the interview texts in the transcripts were coded using open coding, i.e. codes were built and modified throughout the coding process. Some text segments were assigned to more than one code category and text segments that were not relevant for the study objectives were not included in any category. During the process, some of the codes were merged with other codes that had a similar meaning, resulting in 79 codes. One researcher coded the interviews. A second researcher coded two randomly selected interviews to check inter-rater reliability (IRR) [27], calculated as:

$$ IRR=\frac{number\ of\ agreements}{number\ of\ agreements+ disagreements} $$

We found an IRR of 93%.

Codes were grouped into subthemes, which were then grouped into main themes [28]. Four main themes were identified that comprised the allocated codes for all transcripts. No new themes emerged towards the end of the study, suggesting thematic saturation was reached.

The software Atlas.ti version 7.5.6 (Scientific Software Development, Berlin) was used to assist the coding process and extraction of quotes and themes. The quotes presented in this paper were chosen based on their illustration of the described theme or clarifying role of the common or uncommon viewpoints.

## Results

**Fig. 1:**
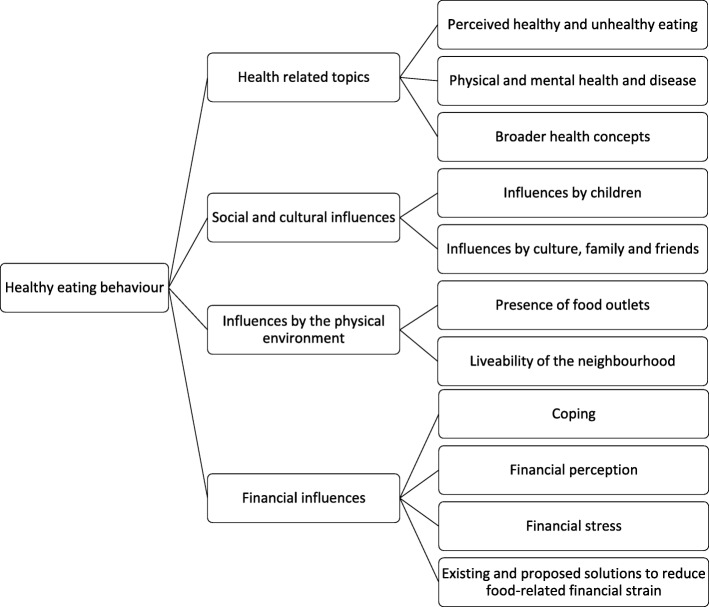
Main themes and their corresponding subthemes

Two males and eight females were interviewed, aged between 35 and 55 years (Table 1). Most participants had an income below the basic needs budget and were lower educated. Six participants were single parents and half of the participants had a paid job. Participants had a Moroccan, Colombian, Surinamese, Curacao, or Polish migration background. Participants were all either overweight or obese, based on their self-reported height and weight. Seven participants were classified as food insecure. The four main themes related to healthy eating behaviour and the corresponding subthemes that were identified in the analyses are described below and depicted in Fig. 1.

**Table 1:** Sociodemographic characteristics of the participants (*n* = 10)

	Age category in years	Sex	Educational level	Household income	Employment status	Marital status	Migration background	Food Bank use	BMI^1^ category	Diet quality score	Food security status
Participant number											
1	45–50	Male	ISCED 2	Below basic needs budget	Currently paid job	Two parent household	Moroccan	No	Overweight	36/ 60	Food insecure
2	40–45	Female	ISCED 2	Below basic needs budget	Currently no paid job	Single parent household	Colombian	No	Overweight	31/ 60	Food insecure
3	45–50	Female	ISCED 2	Below basic needs budget	Currently no paid job	Single parent household	Surinamese	No	Obese Class I (moderately obese)	29/ 60	Food insecure
4	40–45	Female	ISCED 5	Below basic needs budget	Currently paid job	Single parent household	Surinamese	No	Overweight	33/ 60	Food secure
5	40–45	Female	ISCED 2	Below basic needs budget	Currently paid job	Single parent household	Curacao	No	Obese Class I (moderately obese)	41/ 60	Food insecure
6	40–45	Male	ISCED 1	Below basic needs budget	Currently no paid job	Two parent household	Moroccan	Yes	Obese Class I (moderately obese)	35/ 60	Food insecure
7	35–40	Female	ISCED 4	Below basic needs budget	Currently no paid job	Two parent household	Polish	No	Obese Class I (moderately obese)	31/ 60	Food secure
8	50–55	Female	ISCED 1	Below basic needs budget	Currently no paid job	Single parent household	Moroccan	No	Obese Class III (Very severely obese)	46/ 60	Food insecure
9	45–50	Female	ISCED 7	Above basic needs budget	Currently paid job	Two parent household	Surinamese	No	Overweight	32/ 60	Food secure
10	35–40	Female	ISCED 3	Above basic needs budget	Currently paid job	Single parent household	Surinamese	No	Overweight	43/ 60	Food insecure

^1^*BMI* Body Mass Index

### Theme 1. Health related topics

#### Perceived healthy and unhealthy eating

Overall, participants demonstrated relatively good nutrition knowledge; adequate fresh fruit and vegetable intakes were perceived as essential components of a healthy diet. Snacks, fast-food, fatty foods, sugar, and overeating were considered unhealthy. Brown bread consumption was generally considered healthy, in contrast to white bread. Some participants indicated that bread consumption could lead to becoming overweight. Participants had conflicting opinions about whether or not meat consumption was healthy. Some participants considered meat as an essential component of a heathy diet, whereas others considered meat to be very unhealthy.

A frequently mentioned strategy to improve dietary intake was to replace sugar-containing beverages with water. Another strategy to improve dietary intake, and control intakes of unfavourable meal constituents like salt, was home cooking (e.g. making pizza from scratch). Barriers for healthy eating included feeling rushed and pressed for time or tired (e.g. after a working day).

“*Hurry hurry, you know. For example, if you have to go somewhere, for example they have extra lessons in the mosque. Then I notice, quickly baking chips with a minced-meat hot dog and stuff. [ … ] Sometimes you have those empty moments. And then you bake a minced-meat hot dog*.” (Participant 1)

Some participants indicated that healthy cooking and home cooking were difficult and laborious compared to unhealthy cooking and takeaway foods, whereas in the opinion of others healthy cooking was not difficult at all, because healthier cooking techniques (like steaming and oven cooking) were considered easier than less healthy techniques (like frying). Some misconceptions about dietary advice were present, e.g. stating coconut oil as being specifically beneficial for health, while saturated fats like coconut oil are usually not recommended in international and national dietary guidelines [29, 30]. Participants mentioned mostly consulting social media or acquaintances for information regarding healthy eating.

#### Physical and mental health and disease

Most participants clearly linked a healthier diet to chronic disease prevention for themselves and their children.

“*If children eat healthy, they are not ill. Have fewer problems with everything. With concentration too*.” (Participant 7)

Participant 6 really regretted his unhealthy eating pattern in the past, which in his opinion had led to diabetes, and he wanted to prevent that from happening to his children:

“*An example of me. I have always eaten unhealthy and now I have it [disease]. Custard, ice cream, chocolate … [ … ] I should not have done that. But you never knew in advance that you could become a diabetic. If my parents had said that, I would not have done it. But they did not say much. [ … ] They never said ‘that is good and that is bad’. [ … ] It is a pity, but... I did not get it from them*.” (Participant 6)

Another participant became more aware of her lifestyle after being warned by her physician to lose weight in order to prevent cardiovascular diseases and diabetes. One participant mentioned experiencing poorer mental and physical health because of an unhealthy diet and overeating. Contrariwise, poor mental health was seen as a cause of unfavourable eating behaviour. Participants explained they lacked energy to prioritize healthy eating or cooking when feeling unwell, worried, stressed or depressed.

"*Everyone has a difficult situation and you are not in the mood, yes then it is easy to get a bag of fries and throw them in [the frying pan] and everyone has fries. Because it requires fewer actions and if you do not feel mentally well, then washing the dishes is really too much. Going to a supermarket uh, getting out of bed even, is just too much.*" (Participant 3)

#### Broader health concepts

Besides a healthy diet, a healthy weight was considered an important aspect of overall health. Many participants mentioned healthy eating and physical activity as ways to obtain or maintain a healthy weight. One participant felt these factors were interrelated:

“*But I think that if you start exercising, that you, that diet is going to change automatically a little bit*.” (Participant 2)

Some participants mentioned having the intention to exercise more often but not (yet) actually had changed their physical activity level, for example because it was perceived too hard to make time or set one’s mind to it. Costs were not discussed as a barrier for physical activity.

### Theme 2. Social and cultural influences

#### Influences by children

Children played a major role in food choices and food purchases. Participants indicated finding it difficult not to give in to their child’s unhealthy food wishes. Various reasons were indicated for giving in: participants felt sorry for their children if they would not give in, they found it hard to repeatedly reject their child, or they wanted to compensate their lack of time for their child (e.g. due to a busy work schedule) by buying food that the child liked:

“*I work a lot. Night shifts, day shifts and evening shifts. She [child] is alone, I am there with my aunt, but then I felt guilty and then when I left, she started to cry. When I came back I had cookies for her, ‘mommy has brought you cake’. [ … ] You know, or I went to get her at the babysitter and then she said: ‘I missed you, you should not go to work anymore’. ‘That’s okay, mommy will buy a cake for you okay?’*” (Participant 10)

Child food preferences also influenced food purchases and dinner choices. Parents mentioned several strategies to broaden their children’s exposure to and taste for healthy food including: repeated exposure to disliked foods so children could get used to the taste and cooking preferred dishes in a healthier way, such as a homemade pizza rather than store bought or hiding vegetables within a (favourite) dish.

“It’s weird, but they [children] do not want vegetables. But yes, if you for example make chili con carne or for example sauce for spaghetti, then you just throw it through that zucchini. But that is how they eat it.*laughing* So yes, that's how you do it.” (Participant 1)

Setting a good example for their child was mentioned as a motivation for healthy eating by some participants. Further, school food regulations positively influenced child-eating behaviour at school and sometimes also translated into healthier eating behaviours at home. For example, at some schools, unhealthy snacks or drinks were not allowed in class, which also made the children and parents reconsider consuming these products at home. Most participants had a positive attitude towards these school food regulations as they considered it a helpful contribution to adopting healthier eating behaviour.

#### Influences by culture, family and friends

Besides child influences, extended family and friends also influenced eating and food purchasing behaviours. Eating with friends was generally more associated with having a nice time than with healthy eating. Attempts to adopt healthier cooking styles were sometimes hindered by other family members, e.g. when they disliked the lower-salt meals. Eating at family gatherings mostly negatively influenced dietary intake, as family gatherings were often accompanied by unhealthy eating, overeating and sometimes setting bad examples:

“*Well uh, not really influence but they [family] try to force trough their vision or their will and I find that difficult. For example, if I go to my mother, well that she uh thinks he [child] should eat peppers, well, I don’t agree with that.[ … ] After a day at Grandma’s, he [child] goes home and then he ate chocolate, he ate crisps, he ate cake, he ate candy, he ate dinner and preferably ate three other things as well and then also coke and ice cream. Yes, I just think that, I'm really annoyed by that. Really that is just such a frustration.*” (Participant 3)

One participant even decided to limit family visits to reduce her child’s exposure to unhealthy eating habits of the family. Another mentioned strategy was to bring healthy products to these gatherings themselves. Positive influences were also mentioned, as friends and family sometimes served as an exemplary role for healthy behaviour or provided guidance about child upbringing:

“*But the bigger she [child] grew, the more rebellious she became and I say, ‘no, this is not going to happen’. Then I went to talk to my aunt and she coached me a bit and told me I should be strong. No remains no. That’s how I started to learn.*” (Participant 10)

Participants’ cultural background also influenced their eating behaviour, which was reflected in food customs (e.g. providing and consuming large quantities of food at social gatherings) and food choices (e.g. purchasing and cooking traditional foods, mostly indicated to be unhealthy, fatty of sugary foods).

### Theme 3. Influences by the physical environment

#### Presence of food outlets

Participants lived in or near a disadvantaged neighbourhood in The Hague. The presence of sufficient food shops and other facilities in these neighbourhoods was appreciated:

“*Advantages are uhm, yes you can get almost everything here, also from your own culture the groceries. Everything is close by.”* (Participant 3)

The abundance of supermarkets, small food shops (e.g. Turkish shops) and the market were mentioned in this regard. The market was seen as a place to buy large quantities of cheap fruit and vegetables, although some mentioned that these products did not last long enough as they were not fresh. A downside of the abundance of food outlets in the neighbourhood was mentioned to be the food outlets offering unhealthy foods, as participants felt that the presence of these food outlets tempted them into making unhealthy food choices. The food supply at the supermarket checkouts was also considered unhealthy and tempting. Resisting these temptations was especially difficult for children.

“*I also want to leave this neighbourhood. Because [ … ] you cannot blame [name child] because he walks out and it already starts, that Bulgarian there, the fries shop there. I mean, in the morning at around a quarter past eight, he already has fried chicken. Yes, you go with your child to the market to get watermelon, he is twice in the fight at the Kentucky. And then he looks at me like that again [ … ] and then, yes you have to disappoint him. And as a mother you also get tired of that no, no, no [ … ]. So uh sometimes we have a little fight about this too. [ … ] I just want to live somewhere that if you walk out the first ten minutes you will not come across a single snack something. [ … ] this is really too bad for a child.”* (Participant 3)

The school food environment was mostly viewed as healthy by the participants, which is not surprising as most schools adhered to healthy school food regulations. However, as long as the food outlets surrounding the schools offered unhealthy foods, children were tempted to buy those unhealthy foods during the breaks or after school.

#### Liveability of the neighbourhood

Participants had a mostly positive attitude towards their neighbourhoods, for example because of the closeness of shops and facilities, social support of the neighbours, perceived safety, openness towards each other and towards different cultures, and multicultural influences in the neighbourhood. Some negative aspects about the neighbourhoods were mentioned as well, for example noise pollution, dirty streets and perceived lack of safety of the neighbourhood resulting in restricting the child’s outdoor activities.

### Theme 4. Financial influences

#### Coping

Most participants had an income below the basic needs limit and prices were considered important for food purchasing. Various strategies were used to cope with a limited budget, such as careful budgeting and planning, budget-friendly cooking, buying second hand items and buying cheap groceries or groceries on sale. Supermarkets where specific products were the cheapest at that moment were consciously selected, and some participants went to the market around closing time when products were sold for dumping prices. Advantages of planning grocery shopping in advance were firstly preventing buying unnecessary things and thereby saving money, and secondly sticking to healthy eating intentions. Some participants indicated specific financially induced adaptations in their food purchasing behaviour, such as limiting outdoor eating to save money and switching from premium brands to cheaper alternatives of the same products, although the budget products sometimes were perceived less tasty or induced feelings of shame:

“*Yes, I used to be ashamed to buy cheap products [ … ]. I really thought those people would think that I don’t have money. That's how I thought. Some colleagues also said ‘you should not be ashamed, even if all your groceries are premium brands, it's all the same’. It's just another package, just look, it's all the same. I used to buy Cornflakes of 3 euros while I could also get Cornflakes of 1 euro*.” (Participant 10)

Non-basic needs like a holiday with the family or visiting family abroad were important motivators for saving money.

#### Financial perception

Healthy foods (e.g. fruit and vegetables) were perceived to be generally more expensive compared to less healthy foods (e.g. sweets and snacks), making choosing unhealthy options tempting.

“*Well then you go and look and the healthy things are actually really expensive. Yes then you are inclined, [ … ] we better take a sausage roll, you almost want to say that*.” (Participant 3)

Some participants felt discontented about that and indicated that lowering healthy food prices would be a great help in achieving healthier eating behaviour in the population.

“*But the worst help there is are all those sweets in the shops. Those are cheap and the ones that you need are expensive. That is the worst thing they can have. And then some people think: ‘Yes, that is cheap?’ That is why we have a lot of children with obesity here, too many children. Children from 4 years and older, some children are only 5, all teeth are rotten. Wherever you go, [for] 50 cents you have a bag full of candy. You are not going to have a bag full of vegetables for 50 cents. You do not have that. So if you turn that mentality around, it would be better.*” (Participant 5)

However, it was mentioned that using the right strategies (e.g. coping strategies for dealing with a limited budget like buying frozen vegetables) it was possible to buy healthy foods despite having a limited budget. Participants generally felt in control over their grocery shopping behaviour and felt this was not greatly influenced by external factors. Participants demonstrated a conscious attitude towards their financial situation, as reflected in their coping strategies for dealing with a limited budget, knowingly buying products that were a bit more expensive if they lasted longer, and prioritizing basic needs over luxury needs.

#### Financial stress

Despite their generally low incomes, participants overall felt relatively comfortable with their financial situation. As described above, various coping strategies were applied to cope with a limited budget and financial stress. Besides, some participants indicated that money was not the most important thing in their lives. For example, health was considered much more important.

“*For me, money is not everything. For me it is that I can get up every day, that I can breathe every day, that I thank my god. Every day of my life because not everyone can do that and I think that's the best you can do as a person, especially when you get up. Because we cannot buy that, not with any money*.” (Participant 5)

However, as also indicated in the theme about mental health, financial stress was a barrier for healthy eating behaviour, as participant 8 indicated about the time when she was in debt:

“*I did not really buy healthy food then, I just bought what was cheap. I only want to live because you are in the cramp, it’s not possible, it’s difficult*.” (Participant 8)

Regarding basic needs like food and clothes, participants clearly prioritized their children over themselves. For example, participants mentioned to rather skip a meal themselves than that the child would be short on something.

“*I do not care because I prefer [caring for] them [children] rather than myself. I can do with a few slices of bread and peanut butter and then I go to sleep. But they can’t*.” (Participant 6)

Food insecurity was mostly mentioned in reference to the past or to others and not to the participants’ own current experiences, i.e. mentioning past experiences of having insufficient money for food due to debts, or knowing others that were unable to afford sufficient food. Interestingly, participant 1 was classified as food insecure according to the previous questionnaire, but during the interview he specifically mentioned not to worry about going hungry:

“*So, you always have to pay close attention and put everything in order when it comes to finances. For the rest just happy. I mean, my family also. I mean, I'm not worried about, for example, that I'm going to starve, not that*.” (Participant 1)

He made a clear link with the quantity aspect of food security for himself and his family:

“*Healthy eating for me and my family means ensuring that there always is food. Yes. That is first of all healthy, that you have to eat. And secondly, yes, that you pay attention to your diet.*" (Participant 1)

#### Existing and proposed solutions to reduce food-related financial strain

Participants were familiar with several existing resources to reduce financial strain or improve eating behaviour, like several foundations, allowances, debt assistance, dieticians, the Food Bank, and local initiatives. They generally had a positive attitude towards these resources, which were perceived as a welcome helping hand, although some indicated that they would rather not need it. Conceptually the Food Bank was appreciated, but the actual content of the food parcels distributed by the Food Banks was criticized. Participants mentioned that the distributed products were not suitable for preparing a meal and were sometimes rotten or past the expiry date. If bread was provided it was sometimes stale. Suggested improvements for the content of the food parcels were to provide more fresh products like fruit, vegetables, potatoes and other products that can be used to prepare a proper meal. It was further deemed desirable that social contacts would be promoted and facilitated by Food Banks or other organizations, for example by facilitating getting together for a coffee and conversation.

“*The only thing they [Food Banks] don’t have is social contacts*.” (Participant 6)

Other proposed solutions to reduce financial strain and improve dietary habits were providing free meals for those in need, increasing healthy food supply in the neighbourhood (specifically limiting unhealthy snacks at supermarket checkouts and decreasing the number of fast-food outlets) and lowering healthy food prices.

“*What would help me? To eat healthier? If the store prices of those things drop a little, that would be super helpful. Not just for me but for many people*.” (Participant 5)

Barriers for using resources included feeling ashamed, thinking not to belong to the target group, not being eligible for the desired resources, finding it too difficult to register for resources or not knowing where to find the right information. Further, dietary advice provided by dieticians was mentioned to be insufficiently suitable for different cultural backgrounds:

“*For dietary advice, it's just hard in such a neighbourhood as this because you have different cultures. [ … ] I also experienced that at the dietician, yes okay I do get the dietician but I don’t eat all that. And you can’t expect that if it is in your roots not to eat certain things that you just change it*.” (Participant 3)

Several participants felt that resources like Food Banks and allowances were often misused by people who did not need it and that people who actually needed help not always asked for or accepted help.

## Discussion and conclusions

The current study aimed to provide better insight in the needs and perceptions regarding healthy eating among parents living in disadvantaged neighbourhoods in the Netherlands at risk of experiencing food insecurity. Overall, participants showed relatively adequate nutrition knowledge and awareness of the importance of healthy eating behaviour for optimal mental and physical health. Nevertheless, participants indicated various social, environmental and financial barriers to healthy eating behaviour.

### Comparison with previous literature

Consistent with previous research [31], participants acknowledged the importance of healthful eating for chronic disease prevention and overall health. Weight maintenance and child weight maintenance through healthful eating and physical activity was a recurring topic. This finding is in contrast with a previous study [32] that found that participants recognized the importance of improving health habits for themselves but not for their children. Our participants were clearly highly aware of the importance of child weight control, but nevertheless child overweight was a common concern among participants.

Some studies confirm the association between lower nutrition knowledge and lower SES [17, 33] and low (but not very low) food security [34], whereas others indicate adequate nutrition knowledge in these groups [35, 36], which is in line with our findings. Nevertheless, participants generally had a suboptimal diet quality and physical activity level, suggesting that a lack of knowledge was not the driving factor influencing eating behaviour. This is in line with various psychological theories related to health behaviour, all consisting of multiple constructs indicating that a variety of factors influence the eventual health behaviour [37].

Participants voiced several social, environmental and financial barriers to healthy eating behaviour. Social barriers included unhealthy foods offered at social gatherings, bad exemplary roles of others, lacking social support for adopting healthier eating habits, and cultural customs that were associated with overeating and unhealthy food products. Social and family relations are shown to influence eating behaviour [38]. Especially children were noted to play an important role in family food habits [38], which is in line with the views of our participants. Therefore, it is important to consider child influences when developing interventions to improve eating behaviour among families at risk of food insecurity. In line with previous studies [33, 39], lack of time to prepare or cook a meal was another perceived barrier for healthy eating.

Environmental barriers for a healthy eating and lifestyle behaviour included an unfavourable food environment (e.g. an abundance of fast-food outlets). A systematic review on environmental factors and obesogenic dietary intakes showed that the food environment (i.e. less access to supermarkets or greater access to takeaway outlets) was consistently associated with higher overweight prevalence, and mixed results were found for the association between the food environment and dietary behaviours [40]. Living in a disadvantaged neighbourhood may act as a barrier for healthy eating behaviour through increased access to takeaway outlets, thereby increasing the ease of making unhealthy choices [40]. Further, perceived lack of safety was mentioned as a barrier to outdoor activities like physical activity and child outdoor play. Previous research among low-SES women also indicated unsafe neighbourhood environments as barrier for physical activity [41]. Also in line with this study [41], despite the generally low income of this study population and of our participants, costs were not discussed as a barrier for physical activity.

Financial considerations were mentioned as a barrier for healthy eating in two ways. Firstly, some believed that healthy foods were too expensive. Strikingly, this perception will probably only intensify because of the recent national tax increase, which came into force on January 2019 [19]. As the interviews were conducted before January 2019, we were not able to assess the impact of the tax increase on price perceptions and eating behaviour in our study. Therefore, future studies should focus on the effects of the tax increase on eating behaviour, especially in low-SES groups. The perception that healthy foods are expensive is in line with previous studies indicating financial considerations as important barriers for health behaviour among low-SES groups [32, 39, 42–44], although participants were resourceful in finding ways to save money and get healthy foods. Secondly, in line with previous studies [45–47], financial stress and poor mental health were associated with poorer eating behaviour. Interestingly, while most participants had low incomes and 7 participants were previously classified as food insecure (van der Velde LA, Nyns CJ, Engel MD, Neter JE, van der Meer IM, Numans ME, et al: Exploring food insecurity and obesity in Dutch families: a crosssectional mediation analysis, unpublished), participants had an overall positive attitude towards their financial situation and barely mentioned personally experiencing food insecurity at the present. Participants did mention experiencing food insecurity in reference to the past or to others. This might be due to feelings of discomfort or shame when disclosing personal experiences with food insecurity during an interview [48].

To improve healthy eating behaviour among people at risk of food insecurity, participants perceived that changes were needed at the governmental and community and social level. Suggested changes at the governmental level included improving existing resources, for example improving the quality and healthfulness of the Food Bank parcel content. Opposite to the perceptions of our participants, most participants of another Dutch study were satisfied with the food parcels and perceived them as healthy [49], even though their content did not conform to Dutch nutritional guidelines [13]. Another proposed governmental intervention was decreasing healthy food prices. Previous studies consistently show that food taxation and subsidies can effectively improve population dietary behaviour [21], suggesting that subsidizing healthy foods might be a very promising intervention. This makes the recent decision of the Dutch government to increase food taxes [19] highly undesirable. Suggested changes at the community and social level included promoting and facilitating social contacts in the neighbourhood as this was currently lacking according to some participants. The importance of eating in a social context was also highlighted in a previous study among charity-run soup kitchen users [35]. Facilitating social contacts could for example be done at Food Banks by providing a suitable location for social interaction. This might also reduce shame and stigmatization associated with Food Bank use, as this was indicated as a barrier for Food Bank use in previous literature [50, 51] and in our study.

### Methodological considerations

This study deepens the understanding of needs and perceptions of parents at risk of experiencing food insecurity. Our qualitative, open interview approach enabled identifying important themes regarding healthy eating behaviour in this difficult to reach target population. Our analyses confirmed some of the themes that were expected to play a role in healthy eating behaviour based on our previous study and the literature (e.g. family influences) and deepened knowledge on these topics. Additionally, some less anticipated themes emerged during the interviews (e.g. influence of the food environment and importance of social contacts). Our results may not be representative for a national sample of people at risk of food insecurity because we only recruited participants from the current study on food insecurity in disadvantaged neighbourhoods in The Hague, The Netherlands (van der Velde LA, Nyns CJ, Engel MD, Neter JE, van der Meer IM, Numans ME, et al: Exploring food insecurity and obesity in Dutch families: a crosssectional mediation analysis, unpublished). Also, participants volunteered to be interviewed which may have led to a sample with a larger-than-usual interest in nutrition. However, the included participants varied in terms of migration background and other characteristics. Also, thematic saturation for all themes was reached, suggesting that the sample size was sufficient for the aims of our study.

### Implications

Nutrition knowledge and motivation to improve healthy eating behaviour were relatively high among participating parents at risk of food insecurity, yet they indicated various social, environmental and financial barriers to healthy eating behaviour. Therefore, interventions aimed at improving eating behaviour in this unique population should not merely focus on nutrition education but take into account a wider range of social, environmental and financial factors. Because our study population consisted specifically of families with young children living in or near disadvantaged neighbourhoods, the identified themes, barriers and interventions may not be generalizable to other populations at risk of food insecurity. Therefore, future studies are needed to confirm the needs and perceptions regarding healthy eating behaviour in other populations at risk of experiencing food insecurity, e.g. young or elderly populations, childless people, and people with other migration backgrounds. Suggested interventions to improve eating behaviour and reduce food-related financial stain that were identified in our study include facilitating social contacts (thereby potentially enhancing social support for both financial and food-related issues), improving existing recourses (e.g. Food Bank parcel content), culture-specific dietary advice, parenting training focused on handling child food choice influences, and improving the neighbourhood food environment. Also, financial and mental issues should be addressed prior to focusing on improving eating behaviour. Further, possibilities for subsidizing healthy foods or taxing unhealthy foods in the Netherlands should be explored as a potentially promising intervention to improve eating behaviour.

## Supplementary information

**Additional file 1: Table S1.** Food insecurity status assessment.

**Additional file 2: Table S2.** Diet quality score components, dietary guidelines and scoring per component.

**Additional file 3: Table S3.** Topic list and example questions.

## Data Availability

The datasets used and/or analysed during the current study are available from the corresponding author on reasonable request.

## Abbreviations

BMI: Body Mass Index
IRR: Inter Rater Reliability
