# Supplementary material for: Needs and perceptions regarding healthy eating among people at risk of food insecurity: a qualitative analysis
Source: Int J Equity Health. 2019 Nov 27;18:184. doi: 10.1186/s12939-019-1077-0 (PMC6880580; doi:10.1186/s12939-019-1077-0)
Supplement: Supplementary file 1 — Additional file 1: Table S1. Food insecurity status assessment. [file 12939_2019_1077_MOESM1_ESM.docx]

**Additional file 1: Table S1.** Food insecurity status assessment

| **Statement/ question** | |
| --- | --- |
| I (or other family members) worried whether my (or our) food would run out before I (or we) got money to buy more. ^1^ | |
| The food that I (or we) bought just didn’t last, and I (or we) didn’t have money to get more. ^1^ | |
| I (or we) couldn’t afford to eat balanced meals. ^1^ | |
| In the last 12 months, did you (or other adults in your household) ever cut the size of your meals or skip meals because there wasn't enough money for food? ^2^ | |
|  | How often did this happen in the last 12 months? ^3^ |
| In the last 12 months, did you ever eat less than you felt you should because there wasn't enough money for food? ^2^ | |
| In the last 12 months, were you ever hungry but didn't eat because there wasn't enough money for food? ^2^ | |
| In the last 12 months, did you lose weight because there wasn't enough money for food? ^2^ | |
| In the last 12 months, did you (or other adults in your household) ever not eat for a whole day because there wasn't enough money for food? ^2^ | |
|  | How often did this happen in the last 12 months? ^3^ |
| I (or we) relied on only a few kinds of low-cost food to feed my (or our) child/children because I was (or we were) running out of money to buy food. ^1^ | |
| I (or we) couldn’t feed my (or our) child/children a balanced meal, because I (or we) couldn’t afford that. ^1^ | |
| My (or our) child was/children were not eating enough because I (or we) just couldn't afford enough food. ^1^ | |
| In the last 12 months, did you ever cut the size of your child's/ any of the children's meals because there wasn't enough money for food? ^2^ | |
| In the last 12 months, did your child/ children ever skip meals because there wasn't enough money for food?^2^ | |
|  | How often did this happen in the last 12 months? ^3^ |
| In the last 12 months, was your child/were your children ever hungry but you just couldn't afford more food? ^2^ | |
| In the last 12 months, did your child/ any of the children ever not eat for a whole day because there wasn't enough money for food? ^2^ | |

^1^Answer options: Often true/ Sometimes true/ Never true/ I don’t know

^2^Answer options: Yes/ No/ I don’t know

^3^Answer options: Almost every month/ Some months but not every month/ Only 1 or 2 months/ I don’t know
