# Supplementary material for: Needs and perceptions regarding healthy eating among people at risk of food insecurity: a qualitative analysis
Source: Int J Equity Health. 2019 Nov 27;18:184. doi: 10.1186/s12939-019-1077-0 (PMC6880580; doi:10.1186/s12939-019-1077-0)
Supplement: Supplementary file 2 — Additional file 2: Table S2. Diet quality score components, dietary guidelines and scoring per component. [file 12939_2019_1077_MOESM2_ESM.docx]

**Additional file 2: Table S2.** Diet quality score components, dietary guidelines and scoring per component

| **Component** | **Recommendations by the Health Council of the Netherlands^1^ and/ or the Netherlands Nutrition Centre^2^** | **% contribution to component score** | **Units** | **Score** | | |
| --- | --- | --- | --- | --- | --- | --- |
|  |  |  |  | 0 | 5 | 10 |
| Vegetables | Eat at least 200 grams of vegetables daily | 100 | Grams/ d | 0 | *Continuous* | ≥200 |
| Fruit | Eat at least 200 grams of fruit daily | 100 | Pieces/ d | 0 | *Continuous* | ≥ 2 |
| Fish | Eat one serving of fish weekly, preferably oily fish | 50 | Servings/ w | 0 | <1 | ≥ 1 |
|  |  | 50 |  | No fish consumed | Lean or both lean and fatty fish | Mostly fatty fish |
| Bread | Replace refined cereal products by whole-grain products | 50 |  | Mostly white bread | Both white and brown/ whole-grain bread | Mostly brown/ whole-grain bread |
|  | Women: 4-5 brown/ whole-grain sandwiches daily | 50 | Sandwiches/ d | 0 | *Continuous* | ≥ 4 |
|  | Men: 6-8 brown/ whole-grain sandwiches daily |  | Sandwiches/ d | 0 | *Continuous* | ≥ 6 |
| Oils and fats | Replace butter, hard margarines and cooking fats by soft margarines,  liquid cooking fats, and vegetable oils | 50 |  | Butter, hard margarines | Both butter, hard margarines and oils and soft margarines | Oils and soft margarines |
|  |  | 50 |  | Butter on bread or bread is not buttered at all | Semi-skimmed butter or hard margarine on bread | Diet margarine on bread |
| Sweet and savory snacks | For products outside the Wheel of Five: choose an item from the daily selection no more than three to five times per day, and something from the weekly selection no more than three times a week | 25 | Sweet snacks (larger serving)/ w | ≥ 3 | <1 to 2 | Not consumed |
|  |  | 25 | Sweet snacks (small serving)/ d | > 3 | *Continuous* | Not consumed |
|  |  | 25 | Savory snacks (larger serving)/ w | ≥ 3 | 1 to 2 | Not consumed |
|  |  | 25 | Savory snacks (small serving)/ d | > 3 | *Continuous* | Not consumed |

^1^Health Council of the Netherlands. *Guidelines for a healthy diet 2015* (Richtlijnen Goede Voeding 2015). The Hague 2015.

^2^ Netherlands Nutrition Center. *Wheel of Five guidelines* (Richtlijnen Schijf van Vijf). The Hague 2016.
