# Supplementary material for: Needs and perceptions regarding healthy eating among people at risk of food insecurity: a qualitative analysis
Source: Int J Equity Health. 2019 Nov 27;18:184. doi: 10.1186/s12939-019-1077-0 (PMC6880580; doi:10.1186/s12939-019-1077-0)
Supplement: Supplementary file 3 — Additional file 3: Table S3. Topic list and example questions. [file 12939_2019_1077_MOESM3_ESM.docx]

**Additional file 3: Table S3.** Topic list and example questions

| **Topic** | **Example question** |
| --- | --- |
| **General/ introductory topics** |  |
| Birthplace and culture | When and where were you born? |
|  | For how long have you been living in the Netherlands |
| Household composition | What does your family look like? |
|  | Who lives at your home? |
| Living conditions | Where do you live/ which neighborhood? |
|  | What do you think of the neighborhood where you live? |
|  | What kind of house do you have? |
| **Specific topics** |  |
| Healthy eating | What is healthy eating for you? |
|  | What do you think of healthy eating? |
| Skills | What do you think about cooking a healthy meal? |
|  | What do you find easy or difficult when cooking a healthy meal? |
| Influences on eating and food purchasing | What influences how you eat or what kind of foods you buy? |
| Healthy lifestyle | What can you tell about health and nutrition? |
|  | What impact does your health have on what you eat? |
|  | How does your weight affect what you eat? |
|  | How do you think about exercise and health? |
| Eating in a social context | Do other people influence what you eat? Who are they? |
|  | How do they (reference to previous question) affect what you eat? |
|  | What can you tell about eating and coming together with people, for example on parties or social gatherings? |
| Neighborhood | How does the neighborhood where you live affect what you eat? |
|  | What kind of temptations (for you/ for the children) are there in your neighborhood? |
| Cultural influences on eating | What influence does your culture have on your eating habits? |
| Family | What does healthy eating mean for your children (for you as a parent)? |
|  | How important is this (reference to previous question)? |
|  | How do you ensure that your children eat healthy? |
|  | What would you like to teach your children about food and health? |
| Upbringing | What do you find easy or difficult when raising your child? |
|  | What barriers do you experience when raising your child? |
| Financial status | Would you describe your own financial status? |
|  | How do you influence that in daily life? |
| Stress/ financial stress | How does stress affect what you eat or what food you buy? |
|  | What impact do your finances have on how stressed you feel? |
| Food costs | What role does money play in what you eat or what food you buy? |
|  | How do you take into account food costs? |
|  | How do you take into account offers? |
| Priorities | What do you find important or what do you pay attention to when spending your money? |
|  | What do you find important or what do you pay attention to when buying groceries? |
| Nutrition and health | Does eating have an impact on your health? |
|  | How do you notice that (reference to previous question)? |
|  | How do you describe your own health? |
|  | Does your health status influence where or how you buy your groceries? |
|  | Does your physical health prevent you from for example going to the market to buy groceries? |
| Help | What could help you to eat healthier? |
|  | What can people with a limited budget help to have sufficient and healthy food to eat? |
|  | How can the municipality help? |
|  | Which help from the municipality / which foundations do you know? |
|  | What can for example the school or supermarket do to make healthy eating easier? |
|  | What do you think of receiving vouchers to get fruit and vegetables at the market? |
|  | What do you think of the Food Bank? |
| Rearrangement of the neighborhood | If you could rearrange the neighborhood you live in, how would you do that? |
|  | What would you like to remove from the neighborhood or add to the neighborhood? |
| **Closing topics** |  |
| Future | How would you like your future to look like? |
| Unaddressed topics | Is there anything else you would like to address in this interview? |
